# The legacy of the reformasi: the role of local government spending on industrial development in a decentralized Indonesia

**DOI:** 10.1186/s40008-022-00262-y

**Published:** 2022-03-19

**Authors:** Bangkit A. Wiryawan, Christian Otchia

**Affiliations:** Graduate School of International Development, Chikusa-ku Furo-cho 1, Nagoya, 464-8601 Japan

**Keywords:** Decentralization, Capital spending, Local government, Investment, Industrial development

## Abstract

Starting in 2001 the Government of Indonesia employed the Regional Autonomy Law, providing larger fiscal role to the province and district governments. However, our understanding of its impacts on development in Indonesia is still limited. This paper seeks to find the relationship between increasing local governments’ capital expenditure and industrial development with focus in the non-oil and gas sector. Capital spending is thought to have moderation effect on investment, the main channel for industrialization, that should contribute to industrial growth. Our System GMM results suggest that there are positive and significant correlations between capital spending and industrial growth, presenting evidence of local governments’ role. However, we fail to find significant moderation effect between local capital spending and industrial investment towards the sector’s growth. This poses problem for industrialization at the local level. Decentralization progress in Indonesia has been institutionally anchored by the central government, particularly with the introduction of concurrent affairs in 2004 that allowed Jakarta to take a major developmental role in districts and provinces at the cost of lesser local governments’ role. Our study proposes a new institutional model that promote better central–local collaboration.

## Introduction

Decentralization of the government function can be best understood as the devolvement of decision-making from the central government to its lower tier-administration, as described by Litvack and Seddon (1999) in their Briefing Notes for the World Bank. This concept is supposed to bring Pareto growth efficiency according to early decentralization scholars (Tiebout 1956; Oates 1972). These first generation of theorists convinced decision-makers in developed countries and thus propagated a wave of decentralization movement in the 1980s. However, in many developing countries the route to a decentralized governance institution has a quite different context. Large democratic movement in Brazil and the Philippines in the mid-1980s has led to a more decentralized institutions in both countries (Oxhorn et al. 2004). Indonesia followed the same narrative where the centralized-authoritarian rule of then president Suharto met its end in 1998, provided a way for a more democratic bottom-up governance starting in 2001.

**Fig. 1:**
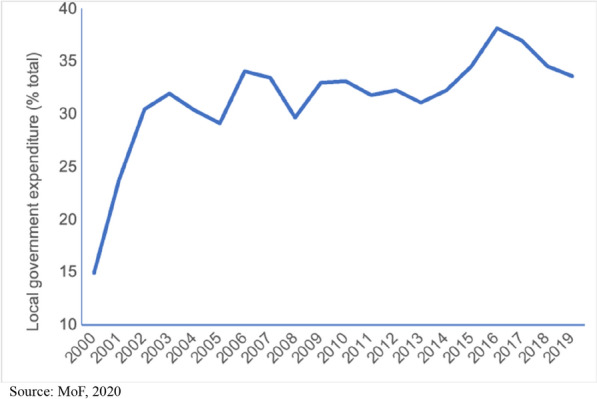
Indonesia’s fiscal decentralization share 2000–2019 (Source: MoF (2020))

Decentralization in Indonesia was part of the nationwide *reformasi*^1^ program. This was done under the background of provincial discontent at around the time (Eaton et al. 2011), specifically in the resource-rich regions. Starting in 2001, provinces and cities/districts are assigned larger fiscal roles that include various governmental affairs excluding executive functions such as foreign and defense affairs. New provinces and cities were also established to meet this demand of a less-centralized state institution. Fiscal decentralization in Indonesia is complimented further with the introduction of direct local elections beginning in 2005. Figure 1 illustrates this change where the share of sub-national against general government spending increased dramatically from 2000 to 2003 during the early phase of decentralization, and then followed by a steady increase until 2017. Consequently, in principle, the sub-national governments are granted larger decision-making abilities.

However, it is important to notice that decentralization transition in Indonesia has not been smooth since the beginning. The transfer of authority from the central to the local government was done in a “big-bang” rush without much preparation and it was directed straight to the lowest level of government, districts and cities, bypassing the province (Nasution 2016). This ill-prepared decision created institutional problems ranging from corruption to the constant lack of capacity building at the local level as pointed out by various studies (Alfada 2019; Henderson and Kuncoro 2004; Kuncoro 2006; Mulyo 2015; Nasution 2016).

Due to the lack of institutional quality at the local level in this first phase, Jakarta slowed down its pace of decentralization with the announcement of Law 32 in 2004. The law introduced the ‘concurrent affairs’ which allowed the active presence of the central government at the sub-national level. Following the law, regulations were set up and various central government’s agencies were re-established in provinces and cities. Some scholars perceived this as an effort to re-centralize political and administrative power (Rahmatunnisa 2015). In between the years, the government has also issued Law 28 on Local Tax that enabled local government to expand their tax base. This law has led to spiking numbers of local regulations in the following years. However, still this does not significantly increase local governments’ capacities especially at the district/city level. Instead, in 2014 Jakarta issued the new decentralization law that (1) maintain the concurrent role of the central government, and (2) provide larger responsibility to the province-level governments at the cost of relatively lower district governments’ role. Despite all the dynamics, in administrative and political sense Indonesia is now a very much decentralized state compared to Suharto’s ‘new order’ era.

It is expected that this dynamic institutional change would bring substantial effect on the economic performance at the local level. Indonesia’s economic progress in the decades prior to this has been hailed as the ‘rising tiger’ due to its unprecedented high growth rate. The progression was largely sustained by the export-driven manufacturing sector, before it was deeply affected by the 1997 Asian crisis. In the subsequent years, the manufacturing share of total GDP had been constantly decreasing, as well as its manufacturing trade specialization (Tijaja and Faisal 2014).

Few studies have explored the link between decentralization on economic development in Indonesia. Setiawan and Aritenang (2019) found a positive impact of decentralization on regional economic growth optimally after 3 years. This confirmed the research of Dartanto and Brodjonegoro (2003), done in the early years of decentralization. They found out that decentralization is positively correlated with local economic growth and the effect is higher in the eastern region. In discussing elite capture, a relevant issue that could affect development under decentralization, Chowdhury and Yamauchi (2010) revealed that village decentralization in Indonesia has not lead to elite capture but rather is representing community’s interests. Contrasting arguments on the impact exists, that highlighted the negative or non-discernible effects of decentralization on the economy (Lewis 2006; Pepinsky and Wihardja 2010; Kuncoro 2006). So far, however, it is still not known whether decentralization can foster industrial development at local level. Industrialization has been regarded as the key towards productive structural transformation in Indonesia and elsewhere (Kniivila 2007; Lin 2010; Otsubo and Otchia 2020). Developing countries employ industrial policy to be able to catch-up with the developed ones. The success of the Newly Industrialized Countries of South Korea, Taiwan, Hong Kong, and Singapore is the famous case of this industrialization strategy by employing export-oriented approach. The same strategy employed by China, through its reform and opening-up (*gaige kaifang*) program in 1978 that paved the way towards an unprecedented sustained growth for multiple decades.

Considering its importance in sustaining growth and promoting structural change, this paper seeks to fill the gap in explaining the impact of decentralization policy on industrial growth at the sub-national level, using Indonesia as the case study. We understand that theoretically, under neo-classical approach, investment serves as the main channel of industrial growth rather than government spending that, in a decentralized sense, is channelled through the local government. Therefore, we also consider the impact of investment; does higher local government spending expand or diminish investment’s effect on industrial growth? What is the possible underlying cause for the relationship to happen?

The rest of this paper will discuss the literature review on decentralization in Sect. 2, then followed by data description and estimation strategy in Sect. 3. Results and discussion are presented in Sects. 4 and 5, respectively. Section 6 concludes my findings.

## Literature review

Arguments that support decentralization and economic growth have been started at least by Tiebout (1956, 1961) and Oates (1972). Tiebout’s mobile consumer model describes how increasing local budget in public service provisions can benefit individual consumers across-jurisdiction by assuming a free-movement setting. This would incentivize local government as they could enjoy higher tax returns. Oates, meanwhile, argues that fiscal federalism provides Pareto-level efficiency as local government is closer to their constituents thus having a deeper knowledge of local economic needs. These theories are influential and were the driving force behind the decentralization wave that occurred starting in 1980s. Subsequent body of works that support this optimistic view include Musgrave (1969), Weingast (1995), and Qian and Weingast (1997).

In a different manner with the optimistic perspectives above, Bahl and Nath (1986) using a sample of 57 developing countries, found that fiscal decentralization happened along with income growth. Thus, they suggested that for developing countries the policy is more appropriate for the middle and upper-middle income one that are richer. This is, however, not the only prerequisite. Wallich et al. (1995) showed that missing institutional framework has been the source of inefficiency in developing-transitional countries of Eastern Europe. Their study was much related to the concern of the institutionalist approach (Acemoglu and Robinson 2013; Acemoglu et al. 2008; North 1991). The lack of institutional quality in accompanying decentralization programs then became the concern of the later decentralization scholars, (Ahmad et al. 2005; Bird and Smart 2002; Azfar et al. 1999; Litvack et al. 1998). Another institutional work includes also Treisman (1999; 2007a) who revealed that there is no conclusively positive impact of decentralization on economic development for developing countries.

Numerous cross-country empirical evidence analyzing the impact of decentralization programs on growth indicators show negative trends (Colombo and Martinez-Vazquez 2019; Davoodi and Zou 1998; Gemmell et al. 2013; Martinez-Vazquez and McNab 2003; Rodriguez-Pose and Kroijer 2009; Ubago-Martínez et al. 2018; Yushkov 2015). Meanwhile, Martinez-Vazquez and McNab (﻿^2006^) despite confirming the negative relationship on growth, found that the effect is offset through its positive impact towards macroeconomic stability, measured by consumer price index.

In the case of Indonesia, Kharisma (2013), Kuncoro et al. (2013), and Kis-Katos and Sjahrir (2017) reveal that decentralization has brought positive impact on development. However, for the latter work, in terms of political decentralization they concluded that there are no positive relationships on the public investment in the education, health, and infrastructure sectors. This means that local expenditures in these sectors do not depend on whether the district governments are democratically elected. Another study found out that local government in the post-2001 period was considered to be negatively affecting business, as argued by Kuncoro (2006). He found that firms failed to grow and descents to a process he called ‘informalization’ or downsizing in order to escape from paying local tax and bribes. Elite capture is another concern that often haunting decentralization process. Firdaus (2018) and Lucas (2016) observed this problem at the village level although the findings of Chowdury and Yamauchi (2010) suggest contrary evidence. Meanwhile Alatas et al. (2013) revealed this capture tendency in a targeted welfare program at the district level.

Further studies also showed that Indonesian decentralization does not bring a positive impact on economic performance (Pepinsky and Wihardja 2010). Their estimation method is unique as it involves the creation of “synthetic Indonesia” in which the country was not decentralized. They attributed the problem to local elite capture. On the other hand, Lewis’s finding (2006) pointed out that the negative relationship is also due to the local government’s tax inefficiency. This led to higher dependence on grant transfers from the central government.

Despite some positive findings, many of the studies on Indonesia reflect the cross-country evidence that fail to establish positive relationship. One of the often cited concern is regarding the low capacity of the local government. This situation has been observed by Wallich et al. (1995), Ahmad et al. (2005), as well as Bird and Smart (2002). The problem limits local government in carrying out developmental role. The theoretical evidence, at least in the case of Indonesia, does not support positive moderation effect from local government on investment that translates to industrial growth. Furthermore, this could imply that different agency, i.e., the central government, is responsible in filling the void.

## Data and methodology

Our analysis uses official data mainly from Statistics Indonesia (Badan Pusat Statistik, BPS). Missing variables due to the unavailability of data are expected when we design this research. The following section discusses data measurement method and descriptive statistics. Then it is followed by elaboration of our estimation strategy.

### Data

Our main data source for the regional data on industry, trade, and inflation are derived from BPS, while investment data are coming from the Investment Coordinating Board of Indonesia (BKPM). We use foreign and domestic investments data on the industry sector. Table 1 presents the descriptive statistics of variables used in the analysis. The total number of observations amounts to 544, covering a time span of 16 years (2004–2019) for 34 provinces. The new province established in 2012, North Kalimantan, is given imputed values to cover for the missing years.^2^

**Table 1:** Descriptive statistics

Variable	Unit	Obs	Mean	Std dev.	Min	Max	Source
MAN	% GRDP	544	14.09	11.48	0.77	67.24	BPS
LCAP	% Total Local Capital Exp.	544	2.94	2.29	0.25	14.21	BPS
FDI_*IND*_	Log Const. IDR million/capita	544	3.64	4.84	0	15.54	BKPM
DDI_*IND*_	Log Const. IDR million/capita	544	3.35	4.90	0	14.82	BKPM
GRDPPC	Log Const. IDR million/capita	544	2.17	0.65	0.70	4.12	BPS
TRADE	% GRDP	544	94.16	46.72	17.53	326.48	BPS
INFLATION	Annual %	544	6.42	4.20	0.02	29.34	BPS

Source: author

We use manufacturing (MAN) as the main proxy for the industry sector. The data are measured by calculating the share of manufacturing value-added in the province level against the total Regional GDP (GRDP) of the said province. Opting to focus more on the labor-intensive activities, we exclude mining activities from the data. This sector is thought to be more capital intensive yet engage in extractive activities that often oppose industrialization (*Dutch disease effect*).

The BPS data source that we use is the yearly statistics of Statistics Indonesia (2007b, 2010b, 2015, 2020c). The record provides information of GRDP by various economic sectors including agriculture and services. The mean value of MAN is 14.09% with a standard deviation of 11.48.

Data of local government spending is used to reflect on the increasing provinces’ and districts’ autonomy. However, we suspect that total local government spending is not the most appropriate measurement in estimating industrial development. Rather it is the capital spending of the local government (LCAP) that is a more appropriate measurement. Largely, this capital expenditure component consists of public investment expenditure such as for infrastructure development, but a lower share yet still a substantial part of the budget goes also for the purchase of building and machinery to support routine civil service activities. The BPS publication source for this data, the yearly Statistics Indonesia (2007a, 2009, 2012a, 2016a, 2020a), and Statistics Indonesia (2006, 2008, 2010a, 2012b, 2014, 2016b, 2018, 2020b), unfortunately, does not separate between the two functions. Therefore, general capital spending is used here instead.

LCAP is measured by calculating the share of province and district government’s capital spending against total local capital spending aggregated at the province level. Table 1 shows that the average share of capital expenditure for each province is at 2.94%. Its standard deviation, which measures the distance between the observation, stands at 2.29%.

Investment data are differentiated based on its origin, whether they are foreign or domestic. The data provided by BKPM are the realization of investment data, meaning that the numbers that went into the real sectors possibly contribute in generating fixed-capital formation in the provinces. Across 23 available sectors in 34 provinces, we selected investment in the non-oil and gas manufacturing sectors. These sectors include food and beverages, textile, pharmaceutical, transport, and machinery. We matched these selected sectors with the manufacturing industry data from the same sectors. Additionally, we also calculate the per capita investment rather than using total value. This way we can control for the dominant effect coming from the advanced region such as Jakarta and Java provinces.

The unit of measurement for these variables is in constant million IDR, using national CPI data as the deflator. Foreign investment data are originally reported in USD, but we adjust it to IDR using mid-yearly official exchange rate issued by BPS. As Table 1 shows, foreign investment data (FDI_*IND*_) has the mean log value of 3.64, with its standard deviation at 4.84. Several provinces at the beginning of decentralization transition phase, up to the end of 2000s received no FDI, especially in the manufacturing sector hence the zero minimum value in the table. Meanwhile, the domestic direct investment (DDI_*IND*_) bears the mean value of 3.35, lower than its foreign counterpart, with a standard deviation of 4.90.

### Estimation strategy

Our estimation approach builds on the framework put forward by Temin (1967) in his work on the pattern of industrial growth with two important points of departure. First, we include a quadratic function of log GDP whereas Temin used a linear specification. Our econometric model also includes additional drivers of industrial development at local level such as local capital expenditure, FDI, DDI, and inflation. Second, the estimation makes use of the System Generalized Method of Moment (GMM), therefore avoiding the problem of endogeneity. This problem is to be expected thus we assume that applying a standard FE-OLS model will lead to a biased result.

Our initial specification under ordinary least square (OLS) regression is as follows:

1 $${\text{MAN}}_{it} = \beta_{1} {\text{LCAP}}_{it} + \beta_{2} X_{it} + \mu_{it} ,$$

where ${\text{MAN}}_{it}$ denotes the non-oil and gas manufacturing share to GDP in province $i$ at year *t*, ${\text{LCAP}}_{it}$ is local government’s capital spending aggregated at province level, $X_{it}$ is a vector of exogenous control variables. $\mu_{it}$ is the ‘fixed effects’ error term.

Endogeneity bias that could affect our *β*_2_ estimate in Eq. (1) may occur from three channels (Lim 2019); measurement error, reverse causality, and unobserved heterogeneity. We address the first issue by strictly using official statistics from BPS and BKPM. This ensure validity and reliability of our data and thus minimize measurement error. Reverse causality is a main concern in the above specification as industrial growth may also affect the size of local governments’ fiscal spending, which run contrary to our argument. In addition, unobserved heterogeneity may add to more bias in our result due to missing variables. As briefly mentioned, only a handful of complete statistical data are available at the sub-national level. Most important unattained variable related to this research is the sub-national spending of central government’s budget.

The common solution to the endogeneity issue is to lag the dependent variable. However, as OLS is not designed to handle “dynamic panel bias” coming from the proposed solution (Roodman 2009), System GMM estimator is used instead. The model was first developed by Arellano and Bover (1995) and Blundell and Bond (1998) as an alternative to the previously developed Arellano and Bond (1991) estimator known as difference-GMM.

The system GMM estimation uses two instrument equations, at the level and the first-difference equation used in the Arellano and Bond method (Roodman 2009). Our specification for system GMM equation is given as follows:

2 $${\text{MAN}}_{it} = \alpha {\text{MAN}}_{it - 1} + \beta_{1} {\text{LCAP}}_{it} + \beta_{2} X_{it} + \mu_{it} ,$$

3 $$\mu_{it} = \eta_{i} + \nu_{it} ,$$

for $i$ = 1,…,*N* and $t$ = 2,…,*T*, with *N* > *T* with $\beta_{1} < 1.$.

${\text{MAN}}_{it}$ is the manufacturing share to GDP in province $i$ at year *t*, ${\text{LCAP}}_{it}$ is local government’s capital spending aggregated at province level, while $X_{it}$ is control variables. $\mu_{it}$ is the ‘fixed effects’ error term that can be decomposed into $\eta_{i} + \nu_{it}$, assuming both are independently distributed across $i$ with the following error component structures (see Blundell and Bond 1998):

4 $$E\left( {\eta_{i} } \right) = 0,\;E\left( {v_{it} } \right) = 0,\;E\left( {v_{it} \eta_{i} } \right) = 0\quad {\text{for}}\;i = 1, \ldots ,N\;{\text{and}}\;t = 2, \ldots ,T,$$

5 $$E\left( {v_{it} v_{is} } \right) = 0\quad {\text{for}}\;i = 1, \ldots ,N\;{\text{and}}\;\forall t \ne s.$$

We also consider the standard assumption of the initial condition of the dependent variable following Ahn and Schmidt (1995) as

6 $$E\left( {{\text{MAN}}_{i1} v_{it} } \right) = 0\quad {\text{for}}\;i = 1, \ldots ,N\;{\text{and}}\;t = 2, \ldots ,T.$$

The orthogonality condition from Eqs. (4)–(6), assuming that there is no serial correlation in the time-varying disturbances $v_{it} ,$ is given as follow

7 $$E\left( {{\text{MAN}}_{it - s} \Delta v_{it} } \right) = 0\quad {\text{for}}\;i = 1, \ldots ,N\;{\text{and}}\;t = 2, \ldots ,T,$$

where $\Delta v_{it} = v_{it} - v_{it - 1}$. The equation can be simplified (Blundell and Bond 1998, p. 118) into

8 $$E\left( {Z^{\prime}_{i} \overline{u}_{i} } \right) = 0.$$

Having instrument of equations at the level and first-difference in the model is thought to bring a risk of instrument proliferation problem. It is a condition where the number of instruments tends to increase exponentially along with the number of time periods used (Heid et al. 2011). This would result in a finite sample bias and will likely to be overidentified. To overcome the problem, following Roodman (2009), we collapse the instrument matrix into single column. In order to check the validity of the model we apply the Hansen test, which is robust to a heteroskedastic condition.

Per capita GDP has thought to be one of the necessary control variables that should be included in a growth estimation model, the other being investment level, population, and human capital (Levine and Renelt 1992). However, as decentralization concept in Indonesia is also based on the population size, having population variable in the model would create a multi-collinearity problem. Applying the human capital variable in the model will also create the same problem. The investment variables, as described in the previous section, are differentiated based on its origin (FDI and DDI) as well as focusing on the industry sector. Other necessary control variables used in the model are trade openness (% GDP), inflation (% annual), and per capita GRDP (GRDPPC) which is measured as log constant of million IDR.

## Results

Our system GMM estimation results are divided into several parts; first we look at the initial impact without covariates before adding in the control variables and the interaction effects between local capital spending data and investments. Following this, we proceed with heterogeneity analysis and robustness check. We bring the findings to formulate our arguments in the discussion part.

### Main results

Table 2 reports the results under System GMM estimation. The one-step estimator is preferred following the argument that its asymptotic variance matrix is found to be more reliable than its two-step counterpart (Blundell and Bond 1998). On the first specification in column (1) shown in Table 2 we can see that the lagged value of the dependent variable is positive and significant. The *β*_1_ coefficient stands below 1, which suffices our assumption in Eq. (1). We can also see that the magnitude and sign are consistent across different specifications. This confirms the dynamic attribute of industrial development in post-reform Indonesia.

**Table 2:** Local capital spending and industrial development: main results

Variables	Dependent variable: manufacturing					
	(1)	(2)	(3)	(4)	(5)	(6)
MAN_(*t*−1)_	0.959*** (0.028)	0.962*** (0.027)	0.959*** (0.026)	0.958*** (0.026)	0.959*** (0.023)	0.961*** (0.023)
LCAP	0.082* (0.043)	0.082** (0.032)	0.083** (0.034)	0.085** (0.036)	0.080*** (0.028)	0.078*** (0.030)
GRDPPC		0.665 (0.822)	0.706 (0.799)	0.727 (0.821)	0.443 (0.660)	0.458 (0.720)
GRDPPC^2^		− 0.143 (0.134)	− 0.153 (0.132)	− 0.156 (0.136)	− 0.132 (0.112)	− 0.116 (0.119)
TRADE_(*t*−1)_			0.001 (0.001)	0.001 (0.001)	0.000 (0.001)	0.000 (0.001)
INFLATION				0.052** (0.024)	0.060** (0.026)	0.057** (0.025)
FDI_*IND*_					0.110*** (0.034)	
DDI_*IND*_						0.057** (0.027)
CONS	0.280 (0.364)	− 0.478 (0.820)	− 0.535 (0.807)	− 0.712 (0.849)	− 0.617 (0.714)	− 0.558 (0.762)
Province FE	Yes	Yes	Yes	Yes	Yes	Yes
Year FE	Yes	Yes	Yes	Yes	Yes	Yes
Observations	544	544	544	544	544	544
Provinces	34	34	34	34	34	34
Instruments	23	25	26	27	28	28
AR-1	0.020	0.019	0.018	0.019	0.020	0.018
AR-2	0.243	0.243	0.241	0.320	0.241	0.365
Hansen (*p*-value)	0.257	0.227	0.222	0.311	0.337	0.356

Source: author

AR-1 and AR-2 denotes Arellano–Bond test with p-values results are reported. They are the necessary diagnostics for dynamic panel data estimation, i.e., GMM

Hansen *J*-test calculates overidentifying restrictions that occurs due to increasing number of instruments. *P*-value is reported

Robust standard error in parentheses, *p < 0.1, **p < 0.05, ***p < 0.01

Coefficient of LCAP is positive and significant, presenting the evidence of local government’s role in promoting the manufacturing sector. Based on this result, we can then interpret that one percentage larger share of province’s capital spending correlates with 0.082 higher share of manufacturing sector. This effect seems to be small by itself but is not unexpected as fixed-capital formation is not the direct contributing factor for industrial growth.

The initial results appear to be well justified judged by the post-estimation diagnostic result. We do not detect serial autocorrelation problems at the AR-2 level and the Hansen *J*-test result shows no sign of overidentifying restrictions as we fail to reject them. This too suggests that the use of instruments in the model is justified.

We then proceed to check the robustness of the main result presented in column (1) to the presence of the covariates. The results show that the magnitude of the target variable changed considerably. For instance, in column (2), we can see that the coefficient of LCAP stays at 0.082 after controlling for per capita GDP. To control for any non-linear relationship between per capita GDP and the dependent variable, column (2) also controls for its squared-term as a covariate. Negative correlations with GRDPPC in some of the specifications in Table 2 suggest a diversion of per capita growth from the industry sector, however it should be noted they are not statistically significant across all specifications.

Furthermore, after controlling for TRADE_(*t*−1)_ and INFLATION in columns (3) and (4), the coefficient of LCAP changes only slightly to 0.083 and 0.085, respectively. The positive sign for TRADE is expected despite its small magnitude and non-significance. It measures both inter-provincial imports and exports of tradable goods, that are dominated by manufacturing products aside of commodities. The positive sign for INFLATION is unique in Indonesian setting, as shown by the studies of Chowdhury (2002) and Winarno (2014).

As we include industrial investment variables into the model, the coefficient of LCAP is getting smaller (column 5 and 6). We suspect that there are some moderation effects caused by these variables that jointly affect the dependent variable. The first investment variable is the foreign investment (FDI_*IND*_) and the second is the domestic investment (DDI_*IND*_). The coefficients for both variables are positive and significant, and especially large for the foreign one. The domestic investment has a considerably smaller coefficient than the foreign one, signifying that industrial development in Indonesia is linked more to FDI compared to DDI.

The interactional effect between LCAP and investment variables is presented in Table 3. Across all specifications, we apply the same control variables used in the previous table. Column (1) shows that despite the positive and significant partial terms of both LCAP and FDI_*IND*_, their interaction terms are insignificant. We also fail to see significant interaction between LCAP and DDI_*IND*_ as shown in column (2). Results shown in column (1) and (2) suffices the post-estimation diagnostics. The AR-2 *p*-value is not significant as well as the Hansen test.

**Table 3:** Local capital spending and industrial development: interaction terms

Variables	All		Non-SAR		Resource rich		Non-imputation	
	(1)	(2)	(3)	(4)	(5)	(6)	(7)	(8)
MAN_(*t*−1)_	0.956*** (0.023)	0.961*** (0.023)	0.961*** (0.020)	0.964*** (0.022)	1.046*** (0.011)	1.042*** (0.012)	0.956*** (0.022)	0.964*** (0.022)
LCAP	0.066** (0.029)	0.057 (0.039)	0.081*** (0.029)	0.070* (0.040)	0.106** (0.047)	0.082** (0.041)	0.070* (0.040)	0.084 (0.058)
FDI_*IND*_	0.103*** (0.039)		0.089** (0.036)		0.216** (0.107)		0.109** (0.043)	
DDI_*IND*_		0.041 (0.030)		0.030 (0.034)		0.090 (0.073)		0.033 (0.029)
LCAP * FDI_*IND*_	0.003 (0.006)		0.004 (0.005)		− 0.021* (0.013)		0.004 (0.007)	
LCAP * DDI_*IND*_		0.006 (0.008)		0.006 (0.007)		− 0.005 (0.006)		0.002 (0.010)
CONS	− 0.654 (0.721)	− 0.554 (0.764)	− 0.813 (0.783)	− 0.809 (0.879)	3.934 (4.825)	− 0.013 (5.070)	− 0.624 (0.750)	− 0.541 (0.804)
Control Var.	Yes	Yes	Yes	Yes	Yes	Yes	Yes	Yes
Province FE	Yes	Yes	Yes	Yes	Yes	Yes	Yes	Yes
Year FE	Yes	Yes	Yes	Yes	Yes	Yes	Yes	Yes
Observations	544	544	464	464	96	96	512	512
Provinces	34	34	29	29	6	6	32	32
Instruments	29	29	29	29	29	29	29	29
AR-1	0.020	0.018	0.032	0.031	0.167	0.169	0.024	0.021
AR-2	0.254	0.390	0.229	0.373	0.954	0.806	0.189	0.292
Hansen (*p*-value)	0.341	0.358	0.385	0.439	1.000	1.000	0.386	0.409

Source: author

AR-1 and AR-2 denotes Arellano–Bond test with p-values results are reported. They are the necessary diagnostics for dynamic panel data estimation, i.e., GMM

Hansen *J*-test calculates overidentifying restrictions that occurs due to increasing number of instruments. *P*-value is reported

Robust standard error in parentheses, *p < 0.1, **p < 0.05, ***p < 0.01

### Additional evidence

To check the strength of the result shown in Table 3 column (1) and (2), subsequent heterogeneity analysis is performed. Firstly, we try to exclude all of the Special Administrative Regions (SARs) from the model. These provinces are Nanggroe Aceh Darussalam (Aceh), Jakarta Capital Region, Yogyakarta Special Region, West Papua, and Papua province. The reason to exclude them is that these provinces have different institutional settings than the others. Their institutional differences vary, which are also influenced by political history and localities.

Jakarta Capital Region differs with other provinces as it does not have a politically elected district government. The city is headed by a Governor who appoints six administrative mayors with little decision-making power. Thus, the administration is more centralized than in other provinces. Secondly, as the capital region, much attention is given from the central government. This contributes to its higher fiscal and human capital resources. Thirdly, historically Jakarta has always been the important economic center of Indonesia.^3^ This further accumulates resources in the area and created large gaps with other urban economic agglomerations in the country.

In the other special regions, distinct institutional setting also exists. In Yogyakarta Special Region, the province is led by a Sultan who held its position hereditary, thus it is not democratically elected, unlike other provinces. Meanwhile in Aceh, the special autonomy given to this region allows them to set up Islamic-based law that is not widely applicable to the other provinces, thus making it difficult to compare. Lastly, in the Papua and West Papua provinces, they enjoyed higher fiscal transfer from Jakarta in the form of General Allocation Grant (DAU) and Special Allocation Grant (DAK), along with a specialized local representative council (Majelis Rakyat Papua) consisted of indigenous people. Like in Aceh and Yogyakarta, these institutional characteristics are not found in the other provinces. Thus, removing these four provinces and Jakarta from the estimations is justified.

After removing the Special Administrative Regions (SAR), we can see in column (3) and (4) of Table 3 that the coefficient of LCAP improved significantly, with the magnitude of the investment variables are getting smaller. The coefficient for FDI_*IND*_ changed slightly from 0.103 to 0.089 while DDI_*IND*_ coefficient change by 0.01. However, despite the partial interaction terms positive and significant for column (3), the treatment effect is not significant. Same argument applies to column (4) showing interactions between LCAP and DDI_*IND*_.

We further check result in columns (3) and (4) by altering the specification, keeping the resource-rich provinces in the model. Those provinces are Aceh, Riau, East Kalimantan, West Kalimantan, Papua and West Papua. The selection is based on the share of natural resources rent against their respective GRDP. The results showed in columns (5) and (6) stated that the coefficient for LCAP improved grealy from the original specifications. The partial interaction terms are still positive and significant for FDI_*IND*_ while it is not for DDI_*IND*_. The interaction terms, however, is negative for both columns, and especially significant for the first one. These results, however, must be carefully interpreted as it suffers from overidentifying restrictions with the implausibly high Hansen test *p*-value result (1.000) for both of the specifications. This might occur due to the now lower number of cross-sectional dimensions that violated the large *N* and small *T* principle for dynamic panel data estimation.

Finally, we extend our analysis by dropping the imputed province and the outlier, which are North Kalimantan and Bengkulu. Dropping these provinces serves double purposes; (1) to increase the initial validity of the model and (2) to justify the use of imputation. The result can be found in Table 3 columns (7) and (8). Coefficient magnitude do not greatly differ from the original specifications with the treatment effect also insignificant. The specifications also suffice the autocorrelation test as well as the overidentification test.

### Robustness check

We estimated our main result on Table 3 using System GMM which is known for its better handling of endogeneity that is coming from both lagged value of the dependent variable and unobserved heterogeneity. Here, we conduct more robustness tests using different estimation methods. The results are summarized in Table 4 in Appendix. In columns (1) and (2), OLS result is presented. We found that LCAP is positive and significant, and so does FDI_*IND*_ and DDI_*IND*_, but similar with our GMM results none of the interactions are significant with similarly small magnitude.

The FE-OLS results on columns (3) and (4) depart from the previous model, showing partial significance only for FDI_*IND*_ but the full treatment effects are negative and insignificant. Employing Difference GMM, as shown in columns (5) and (6) also do not yield significant results. Table 4 shows that our findings are not unique to a specific method.

## Discussion

Results show that local capital expenditure, by itself, has a positive impact on regions’ industrial growth. Districts and provinces with larger share of capital spending have a higher marginal effect on the sector’s growth. However, simply looking at this relationship without addressing the more important channel of industrialization would result in poor interpretation. Therefore, this research assessed the impact of investments in the sector, and its interaction with local capital spending. The negative albeit insignificant interaction terms across the main specifications suggest that industrial growth could be slower in provinces with high local capital expenditure and high levels of FDI or DDI. These findings are quite puzzling and merit additional discussion.

The results brought up earlier discussions raised by Nasution (2016) that highlighted the weak capacity of the local governments in the case of Indonesia, and the studies of Bird et al. (1995) in the case of democratic-transitional countries. In this case, they have not been able to provide efficient public service to channel private investment for industrial growth. This is an issue that has also been explored previously (see Lewis 2006; Mulyo 2015; Pepinsky and Wihardja 2010).

The weak capacity of the local government has been an inherent problem in Indonesia. There are two sides of this issue. Firstly, decades-long centralized administration under Suharto has impaired much of local institutional capacity. His 1974 decentralization initiative was merely administrative, with political power still largely residing around him. Given this condition, the sudden liberalization of province and district governments following the 1999 Regional Autonomy law has failed to encourage investment growth. Increasing responsibilities that come with fiscal and political decentralization outweigh their capacities and capabilities. Thus, promoting development through structural transformation has not been the primary option of the local leaders. Consequently, their ability to expand their revenue base is limited as productivity was low, which in turn limit their development role especially in the early period of reform (2001–2004).

Under these circumstances, the local government resorted to Jakarta for assistance. Their budgetary structure is composed of large transfer allocated by the Ministry of Finance. At the district level, the share of central government transfer to their own budget was as much as 90 percent in 2001. Meanwhile, their own-source revenue constitutes only 7 percent of their revenue (Nasution 2016).

Secondly, at the same time the central government resolved this capacity gap in the implementation level by introducing the ‘concurrent affairs’ with the 2004 law on Regional Government, and further strengthened in 2014. This paved the way for the reintroduction of central government programs and agencies down to the district level. Currently, most of the strategic infrastructure projects lies within the domain of the central government, or a joint-cooperation between the central and the local government. For the proponent of regional autonomy in Indonesia, this was viewed as a ‘recentralization’ effort (Rahmatunnisa 2015). However, this move has been in line with previous theories addressing the lacking capacity of local government (Ahmad et al. 2005; Bird and Smart 2002). On the one hand, this new institutional arrangement has contributed to the substantial increase, albeit still limited, of sub-national revenue from 11.8 to 19.7 percentage share of GRDP between 2004 and 2019. However, on the other hand, this significant increase in revenue is not reflected on the expenditure side. Local capital spending only increased at merely 1.15 percent from 2.2 in 2004 to 3.35 percentage share of GRDP in 2019.

The dramatic increase in revenue reflects the growth on the productive sector that goes into the provinces’ and districts’ balance through taxes. However, the marginal increase in capital spending serves as a testament on the limited role of local government in promoting industrial development which owes to the two factors described above. Barring other things, capital spending is necessary to facilitate investment through the generation of fix-assets such as roads, bridges, ports, and others in return for larger industrial output.

Another factor worth noting in Jakarta’s decision to make large intervention at the local level is the concern over elite capture. This is quite a common phenomenon in developing countries underwent democratic reform such as in Russia (Blanchard and Schleifer 2001). In Indonesia, the problem has been highlighted by several studies (Firdaus 2018; Lucas 2016; Alatas et al. 2013). This would further prevent capacity building by the district and province governments, thus justified the ‘recentralization’ attempt by the central government.

To sum it up, in a decentralized Indonesia after the ‘*reformasi*’, the role of the local government in carrying out development project has been limited by both their lacking of institutional capacity as well as the larger presence of the central government. In terms of industrial development, the absent of significant positive interaction terms across all specifications in Table 3 demonstrate this tendency. Although it is not shown in our estimation tables due to the missing variables, we suspect that larger moderation effect on private investment is coming from the central government. Jakarta’s enormous effort on infrastructure development, especially in the last two administrations of Susilo Bambang Yudhoyono and Joko Widodo, contributed to the sustained growth from 2004 to 2019. The positive relationship between local capital expenditure and industrial growth is perceived as a complimentary effect of central government’s program.

Improving the relationship between public and private investment for the benefit of industrial development will remain a major challenge. This burden might be too big of a task for district governments to handle, and less of a challenge for the provinces which has better resources. However, there are several steps that could serve as the alternative to improve local government’s efficiency in this area. First, Jakarta needs to reformulate their ‘concurrent affairs’ strategy. The current strategy of central-local collaboration often does not require substantial role of the local governments, thus has not been able to nurture capacity development effectively, as suggested by our findings. In most cases, local government involvement is limited to the pre-planning and planning stage and much less in the implementation stage. A larger role in the implementation stage would force them to expand their capacity further while at the same time this would reduce the over-dependency issue.

Secondly, from the top-down side Jakarta needs to reduce their direct role at the local level in favor of facilitating and providing assistance to their local counterparts. They should remain responsible for the national strategic development projects and the promotion of inter-provincial linkages, but should not spearhead local development. Facilitation and assistance could be provided through the central government offices located in cities or districts.

Lastly and of no less importance is to prevent elite capture through strengthening institutional/regulatory framework. Capture would discourage capacity development and hindered structural transformation process. This has been one of the concern of Jakarta in the past. Capable local governments would prioritize the development of productive sector.

In order to make this plan work, significant institutional change that alters the “concurrent affairs” concept would need to be carried out. The new collaboration model should empower local governments more. In the long run this would nurture their capacity to better link with industrial investment and thus push for productive structural transformation. This alteration would require political will from all key stakeholders, particularly the central government who must provide all necessary means.

## Conclusion

Following its major economic and political ‘*reformasi*’ in 1998, the Government of Indonesia introduced the Regional Autonomy bill in 2001 that effectively increased the role of the local governments. This paper attempts to unveil the impact of provinces’ and districts’ expenditure on industrial development. To this end, we extend our analysis by exploring its relationship with foreign and domestic direct investments. Using province and district-aggregated data, the System GMM estimation revealed that larger local capital spending correlates positively with the industry sector. This finding is robust across different specifications. However, we fail to find positive and significant interactions between local capital spending and investment. If anything, the result showed a negligible effect. This suggests that with every percentage increase of local governments’ capital spending, the impact of foreign and domestic investment does not increase accordingly. It is suspected that larger positive impact that channeled investment on industrial growth is coming from the central government’s side.

In a decentralized Indonesia, issues regarding local government capacity remained a major institutional challenge. The decision to push for deep decentralization in the early period (2001–2004) exacerbated this situation, lead to Jakarta’s intervention through the second decentralization law that introduced ‘concurrent affairs’. This intervention, in turn, created over-dependency towards the central government, resulting in the negative interaction effect in our finding. To improve this condition, we argue that the central government needs to formulate a new institutional strategy that allows for larger empowerment of its local counterparts. The current model gives too dominant role for the central government in economic affairs, i.e., infrastructure development, and the function is carried out separately between central and local. The collaborative strategy is also meant to improve local governments’ capacity through budget reallocation and Jakarta’s assistance in development projects. Achieving this, however, requires political will especially from the central government who need to incrementally devolve their function to the local government assuming a certain institutional quality has been met. Future research could compliment the finding in this chapter by studying the distributional impact of the central government’s expenditure towards industrial development.

## Data Availability

Data are available upon request to the corresponding author.

## Appendix

See Tables 4 and 5.

**Table 4:** Robustness check

Variables	OLS		FE-OLS		Difference-GMM		System-GMM	
	(1)	(2)	(3)	(4)	(5)	(6)	(7)	(8)
MAN_(*t*−1)_	0.965*** (0.014)	0.969*** (0.014)	0.888*** (0.025)	0.889*** (0.025)	0.871*** (0.085)	0.845*** (0.094)	0.956*** (0.023)	0.961*** (0.023)
LCAP	0.042* (0.024)	0.038 (0.030)	0.020 (0.033)	− 0.010 (0.042)	0.123* (0.061)	0.096* (0.052)	0.066** (0.029)	0.057 (0.039)
FDI_*IND*_	0.082*** (0.027)		0.103** (0.040)		0.090* (0.053)		0.103*** (0.039)	
DDI_*IND*_		0.076* (0.040)		0.031 (0.047)		− 0.032 (0.044)		0.041 (0.030)
LCAP * FDI_*IND*_	0.007 (0.008)		− 0.021 (0.014)		− 0.001 (0.007)		0.003 (0.006)	
LCAP * DDI_*IND*_		0.007 (0.007)		− 0.013 (0.011)		0.006 (0.006)		0.006 (0.008)
CONS	− 0.597 (0.469)	− 0.419 (0.465)	2.107*** (0.605)	2.086*** (0.609)			− 0.654 (0.721)	− 0.554 (0.764)
Control Var.	Yes	Yes	Yes	Yes	Yes	Yes	Yes	Yes
Province FE	No	No	Yes	Yes	Yes	Yes	Yes	Yes
Year FE	No	No	Yes	Yes	Yes	Yes	Yes	Yes
Observations	544	544	544	544	544	544	544	544
Provinces	34	34	34	34	34	34	34	34
Adj. *R*^2^	0.985	0.985	0.824	0.823				
*F*-stat.	4809***	4134***	2464***	1599***				
Instruments					29	29	29	29
AR-1					0.013	0.013	0.020	0.018
AR-2					0.232	0.321	0.254	0.390
Hansen (*p*-value)					0.716	0.764	0.341	0.358

Source: author

AR-1 and AR-2 denotes Arellano–Bond test with *p*-values results are reported. They are the necessary diagnostics for dynamic panel data estimation, i.e., GMM

Hansen *J*-test calculates overidentifying restrictions that occurs due to increasing number of instruments. *P*-value is reported

Robust standard error in parentheses, **p* < 0.1, ***p* < 0.05, ****p* < 0.01

**Table 5:** Correlation matrix

Variables	MAN	LCAP	FDI	DDI	GRDPPC	TRADE
MAN	1					
LCAP	0.18***	1				
FDI_*IND*_	0.46***	0.24***	1			
DDI_*IND*_	0.33***	0.28***	0.47***	1		
GRDPPC	0.28***	0.47***	0.46***	0.41***	1	
TRADE	0.27***	0.14***	0.29***	0.24***	0.45***	1
INFLATION	− 0.03	− 0.01	− 0.21***	− 0.20***	− 0.09**	− 0.04

Source: author

**p* < 0.1, ***p* < 0.05, ****p* < 0.01

## Abbreviations

BKPM: Badan Koordinasi Penanaman Modal (Investment Coordinating Board)
BPS: Badan Pusat Statistik (Statistics Indonesia)
DAU: Dana Alokasi Umum (General Allocation Grant)
DAK: Dana Alokasi Khusus (Special Allocation Grant)
DDI: Domestic direct investment
FDI: Foreign direct investment
GMM: Generalized method of moment
GDP: Gross Domestic Product
GRDP: Gross Regional Domestic Product
GRDPPC: Gross Regional Domestic Product per capita
OLS: Ordinary least square
SAR: Special Administrative Region

## Footnotes

1 *Reformasi* is the Indonesian style of democratic and market reform, employed as a counter-narrative to the authoritarian and crony-capitalism characteristics of the Suharto administration that lasted for over three decades. This concept deals directly with decentralization issue as it constitutes one of the six main agendas of *reformasi*.

2 Our imputation method is based on Rubin’s (1987, 1996) multiple imputation method. It is a predictive approach for handling missing data in a multivariate analysis and it uses both classical and Bayesian techniques (Zhu 2014).

3 Jakarta’s prominent status as economic and administrative center has its historical context. In the early seventeenth century, the Dutch East Indies Company (VOC) moved their trading post from the more prosperous Banten region to the neighboring Jakarta bay area, as the Banten Empire weaken, Jakarta emerge as the region’s economic hub. The later Dutch Colonial Government and the present Indonesian Government continued to base their administrative and economic activities on the city.
